# Evaluation of Band-Selective HSQC and HMBC: Methodological Validation on the Cyclosporin Cyclic Peptide and Application for Poly(3-hydroxyalkanoate)s Stereoregularity Determination

**DOI:** 10.3390/polym10050533

**Published:** 2018-05-16

**Authors:** Elsa Caytan, Romain Ligny, Jean-François Carpentier, Sophie M. Guillaume

**Affiliations:** Univ Rennes, CNRS, Institut des Sciences Chimiques de Rennes—UMR6226, F-35000 Rennes, France; romain.ligny.1@univ-rennes1.fr (R.L.); jean-francois.carpentier@univ-rennes1.fr (J.-F.C.); sophie.guillaume@univ-rennes1.fr (S.M.G.)

**Keywords:** 2D NMR, HSQC, HMBC, band-selective, tacticity determination, stereoregular polyesters

## Abstract

Band-selective (bs) HSQC, improving spectral resolution by restriction of the heteronuclear dimension without inducing spectral folding, has been recently used for polymer tacticity determination. Herein is reported an evaluation of various bs-HSQC and bs-HMBC sequences, first from a methodological point of view (selectivity, dependence to INEPT interpulse delay or relaxation delay), using the cyclic peptide cyclosporin selected as a model compound, and then from an applicative approach, comparing tacticity determined from bs-HSQC and bs-HMBC experiments to the one obtained from 1D ^13^C{^1^H} on poly(3-hydroxyalkanoate)s samples. For HSQC sequences, the ^13^C selectivity scheme consisting in substituting a ^13^C broadband refocalization by a selective one revealed itself problematic, with unwanted aliased signals, whereas the insertion of double pulsed field gradients spin-echo (DPFGSE) or the use of opposite sign gradients bracketing a selective refocalization gave satisfactory results. Determination of the probability of syndiotactic enchainments, *P_s_*, by bs-HSQC is fully consistent and no precision loss was observed when decreasing acquisition time (37 min vs. 106 min for 1D ^13^C{^1^H}). Bs-HMBC, although not straightforwardly applicable for tacticity determination, could provide (after a calibration step) an alternative for compounds of which only ^13^C carbonyl signals are resolved enough for discriminating between *syndiotactic* and *isotactic* configurations.

## 1. Introduction

Since the early 1980s, microstructural control during polymerization reactions has gained much attention and more recently the concept of sequence controlled polymers has appeared [1,2,3]. The control of tacticity or of monomer sequence in the polymer chain can lead to unprecedented properties (thermal, mechanical, solubility, self-assembling, etc.) and allows access to diversified, valuable, sometimes unique polymer materials.

Stereoselective ring-opening polymerization (ROP) of chiral cyclic esters is one of the most effective routes for the synthesis of stereoregular or monomer sequence-controlled polyesters. Initially, much work has been devoted to the ROP of *racemic* lactide (the cyclic dimer of lactic acid) to obtain polylactide (PLA)—an alternative to common petrochemical-based plastics—with a variety of microstructures: *heterotactic*, *isotactic*, or stereoblock enchainments [4,5,6,7,8,9,10,11,12,13].

**Scheme 1:**
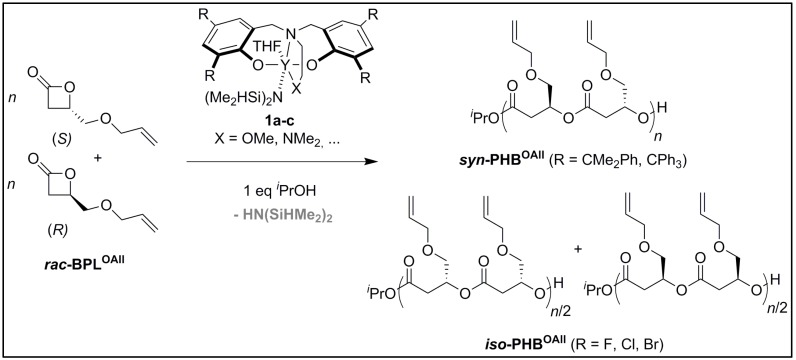
Synthesis of *syndio*- or *iso*-PHB^OR^s from the ROP of *racemic* BPL^OR^s with Y{ONXO^R2^}(N(SiHMe_2_)_2_)(THF)/*i*PrOH catalyst systems, illustrated with the ROP of *rac*-BPL^OAllyl^.

ROP of β-lactones is much less documented because this family of four-membered cyclic esters is inherently more reluctant to ring open and even more in a stereoselective fashion [14]. Some of us have shown that yttrium catalysts stabilized by non-chiral tetradentate amino-alkoxy or diamino-bisphenolate dianionic ligands {ONXO^R2^}^2−^ (X = O, N) (Scheme 1) can provide high chain-end-stereocontrol during polymerization of *racemic* mixtures of such chiral β-lactones. Applying these catalysts to different families of β-lactones revealed different stereoselective abilities (*iso-*/*syndio-*selectivity) simply by changing the nature of the R substituent at the *ortho* position of the phenolate ligand. In the ROP of *racemic* β-butyrolactone, high probability for *syndiotactic* enchainment (*P*_s_) in the resulting poly(3-hydroxybutyrate) has been achieved upon using sterically bulky aryl-type substituents (R = CMe_2_Ph or CPh_3_; *P*_s_ = 0.91 and 0.95, respectively) [15,16,17,18]. On the other hand, for the ROP of *racemic* alkyl β-malolactonates (MLA^R’^s, R’ = allyl (All) or benzyl (Bn)), *syndio*selectivity in the main chain of poly(β-malolactonate)s has been observed specifically upon using *ortho*-chlorinated phenolates (*P*_s_ > 0.95) [19,20]. More recently, for the ROP of another family of β-lactones, namely *racemic* 4-alkoxymethylene-β-propiolactones (BPL^OR’^s, R’ = Me, All or Bn), we evidenced that bulky aryl-containing R *ortho*-substituents in the phenolate ligand also induce high *syndio*selectivity (*P*_r_ > 0.90) in the resulting alkoxy-functionalized poly(3-hydroxyalkanoate)s (PHB^OR^s) (Scheme 1). More remarkably, an unprecedented complete switch of the catalyst selectivity from *syndio*- to *iso*-selectivity (*P*_i_ > 0.90) was observed upon using *ortho*-halogenated ligands [21].

The chemical shifts of some carbons in a polymer chain are sensitive to the relative stereochemistry of nearby stereocenters. This proves to be of particular importance for investigating the microstructure of stereoregular polymers since it allows the observation of the different monomer sequences because the respective resonances are split. Tacticity of the synthetic polymers is mostly assessed by ^13^C{^1^H} NMR because of the large chemical shift dispersion. Unfortunately, ^13^C{^1^H} NMR spectroscopy requires long experiment times to give spectra with adequate signal-to-noise ratio (SNR). Consequently, ^1^H-detected 2D heteronuclear NMR experiments such as HSQC or HMBC enable reducing the acquisition time provided that ^13^C spectral resolution can be kept high enough for resolving the different ^13^C signals [22].

Band-selective (bs) heteronuclear 2D NMR experiments were first introduced to enable restriction of the heteronuclear chemical shift domain (thereby improving the spectral resolution) without inducing spectral folding [23,24,25]. Improved spectral resolution found applications in structure elucidation of compounds exhibiting crowded spectral regions, such as peptides [23,24,26] or oligosaccharides [25,27,28,29,30]. More recently, bs-HSQC has been used for studying ^35,37^Cl isotope shift for ^13^C [31,32,33,34] and for quantifying purposes; its improved sensitivity (compared to direct ^13^C acquisition) allows studying the relative abundance of triad signals in polyacrylonitrile samples [22] or even isotopomers signals in halogenated natural products [33].

^13^C selectivity was first obtained using “soft pulses”: soft 90° or 180° ^1^H pulses (low power rectangular pulses), manipulating only Hα in peptides and observing C(=O) [23,24], or soft 180° ^13^C pulse (DANTE pulse train) in the first INEPT step of HSQC, transferring magnetization only to the desired ^13^C [25]. After 1993, ^13^C soft pulses (DANTE pulse train, Gaussian or REBURP shaped pulses) were combined with ^1^H spin-lock and adequate phase cycling to obtain the desired ^13^C selectivity [27,28,30]. Opposite sign gradients were used in 1995, bracketing a selective 180° refocalization pulse [35], and this selectivity “sandwich” was implemented in numerous homonuclear or heteronuclear bs experiments. Pulsed Field Gradient Spin-Echoes (PFGSE) were first introduced for solvent signal suppression [36,37] and were used in bs-heteronuclear experiments, either for direct ^13^C selectivity in HSQC or HMBC experiments [26,29], or for elimination of unwanted peptide protecting group resonances (DPFGSE applied on the ^1^H region of protecting groups) [38]. Recently, band-selectivity in HSQC-related experiments was obtained using highly selective shaped pulses (Gaussian cascade [39,40]) for ^13^C refocalization during the reverse-INEPT step in place of the adiabatic pulses used for uniform broadband refocalization [31,32,33,34], or using them in a DPFGSE inserted in the HSQC sequence [22].

Herein, we describe an evaluation of bs-HSQC and HMBC experiments as quantitative tools. In a first part, cyclosporin was used as a model compound for studying the quantitative response of bs-HSQC and HMBC variants when varying experimental parameters: influence of ^13^C selective pulse offset, INEPT interpulse delay or relaxation delay were observed. In a second part, these bs experiments were used on poly(3-hydroxyalkanoate)s (PHB^OR^s) samples for tacticity determination, which is commonly determined by 1D ^13^C{^1^H} spectra. The decrease of the experimental time when using bs-HSQC methods instead of 1D ^13^C{^1^H} did not alter the precision or values of *P*_s_ (probability of *syndiotactic* enchainment between BPL^OR^ units). Bs-HMBC methods could be used when the carbonyl region is the only one presenting enough signal dispersion, but verification is still to be made concerning the linear relation between *P*_s_ value obtained by HMBC and 1D ^13^C{^1^H} on the carbonyl region.

## 2. Materials and Methods

### 2.1. NMR Acquisition Parameters

1D and 2D NMR spectra were recorded on a 500 MHz Bruker Av III HD spectrometer (Billerica, MA, USA), fitted with a direct broadband 5 mm probehead (BBO) carefully tuned on both ^1^H and ^13^C channels. Sample temperature was set to 298 K. The cyclosporin sample was a sealed sample containing 50 mg of cyclosporin A in 0.5 mL of C_6_D_6_. Polymer samples were prepared by dissolving about 35 mg of solid in ca. 0.5 mL of CDCl_3_. 1D ^13^C spectra were acquired using Bruker standard zgpg30 sequence (30° flip angle, bilevel ^1^H Waltz-16 decoupling). Acquisition time was 1.05 s and relaxation delay D1 2.00 s. Bs 2D HSQC and HMBC spectra were acquired using following parameters: ^1^H spectral window was 9.2 or 8.0 ppm for cyclosporin and polymer samples, respectively, resulting in acquisition time of 0.44 or 0.51 s, respectively (TD 4096). ^13^C spectral window was 12 or 4 ppm for cyclosporin and polymer sample, respectively, and 256 t_1_ increments were used. For HSQC spectra, broadband decoupling during acquisition was obtained with the standard GARP scheme, and unless otherwise specified interpulse INEPT delay was optimized for a 145 Hz J value for the cyclosporin sample and 132 Hz for polymer samples. For both HSQC and HMBC spectra, unless otherwise specified, relaxation delay D1 was set to 1.5 s.

For HSQC experiments, five sequences were used (Supplementary Materials, Figure S1):

- Standard Bruker hsqcetgpsisp2.2 (from which the following are derived);
- Standard Bruker shsqcetgpsisp2.2: first ^13^C adiabatic refocalization pulse was replaced by a selective 180° refocalization pulse bracketed by gradients of opposite sign [35];
- hsqcetgpsisp2.2-REFOC: ^13^C adiabatic refocalization of the reverse INEPT was replaced by a selective 180° refocalization pulse [31,32,33,34];
- hsqcetgpsisp2.2-DPFGSE: a double pulsed field gradients spin-echo (DPFGSE) using selective 180° inversion pulse was added after the t_1_ evolution period [29]; and
- hsqcetgpsisp2.2-DPFGSESYM: one PFGSE using selective 180° inversion pulse was inserted before the t_1_ evolution period and another one after t_1_ evolution period [29].

For HMBC experiments, three sequences were used (Supplementary Materials, Figure S2):

- Standard Bruker hmbcctetgpl2nd (from which the following are derived);
- Standard Bruker shmbcctetgpl2nd: ^13^C adiabatic refocalization was replaced by a selective 180° refocalization pulse; and
- hmbcctetgpl2nd-SPFGSE: a single pulsed field gradient spin-echo (SPFGSE) using selective 180° inversion pulse was inserted after the t_1_ evolution period [26,29].

^13^C selective 180° pulses were designed using the Q3.1000 Bruker standard shape [39], covering a 600 Hz bandwidth, resulting in pulse duration of 5.747 or 5.683 ms for refocalization or inversion, respectively (durations calculated in the Bruker Shapetool program). Pulse powers were calculated in the Bruker Prosol program.

### 2.2. NMR Spectra Processing

1D and 2D spectra were processed using Topspin software (Bruker Biospin, Billerica, MA, USA). 1D ^13^C spectra were processed using zero-filling from 64 to 128 k, line broadening 1 Hz, manual phase correction and five-degree polynomial baseline correction on the whole spectral window. 2D spectra were processed using zero filling from 256 to 1024 in F1 dimension, squared sine-bell apodization (SSB 2) in both dimensions, manual phase correction (for HSQC only since HMBC are magnitude mode spectra), and five-degree polynomial baseline correction on the whole spectral window in both dimensions. HSQC spectra acquired using the hsqcetgpsisp2.2-DPFGSESYM sequence had to be reversely Fourier-transformed in the F1 dimension due to the presence of a ^13^C inversion pulse before t_1_ evolution period which inverts the apparent evolution frequency of ^13^C magnetization [29].

1D and 2D peaks integration was performed using MNova software (Mestrelab Research, Santiago de Compostela, Spain). Integral regions were saved so that integration was always performed with the same settings for a given signal.

### 2.3. Polymerization Procedure

The PHB^OR^ samples used for tacticity determination were synthesized according to the reported literature [21].

## 3. Results

**Scheme 2:**
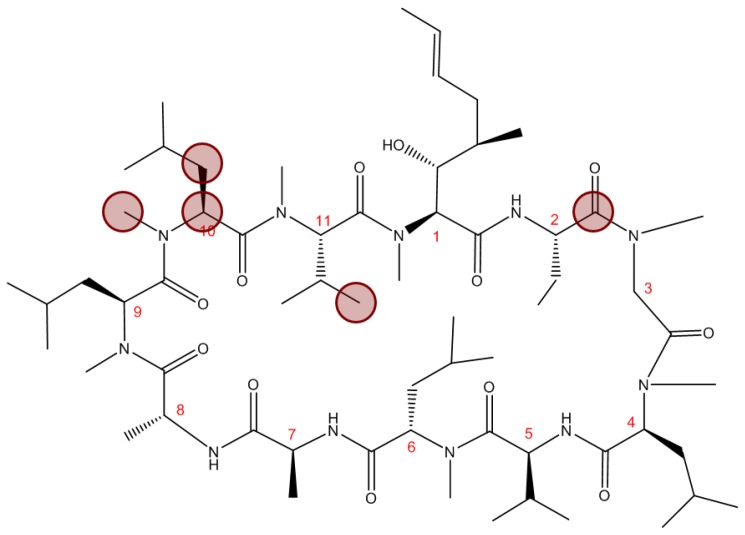
Cyclosporin molecule with selected signals highlighted: Abu-CO, CH α Me-Leu 10, CH_2_ β Me-Leu 10, CH_3_N Me-Leu 10 and CH_3_ γ Me-Val 11.

The methodological study on cyclosporin (Scheme 2) consisted in evaluating the influence of several NMR acquisition parameters on the integral value of HSQC/HMBC peaks. Repeatability of the whole measurement (NMR spectrometer settings, data acquisition, process and integration) has been evaluated for the four bs-HSQC sequences (Table 1), indicating a good precision for all of them (less than 3% standard deviation), and even better for hsqcetgpsisp2.2-REFOC (less than 2%).

For the cyclosporin sample, a few representative peaks (Scheme 2) were chosen to study the influence of carbon multiplicity, T_1_ or ^1^*J*_CH_, on the integration value (Table 2). For the following studies, measurements were not replicated.

### 3.1. Spectra Quality: Selectivity and Aliasing

**Figure 1:**
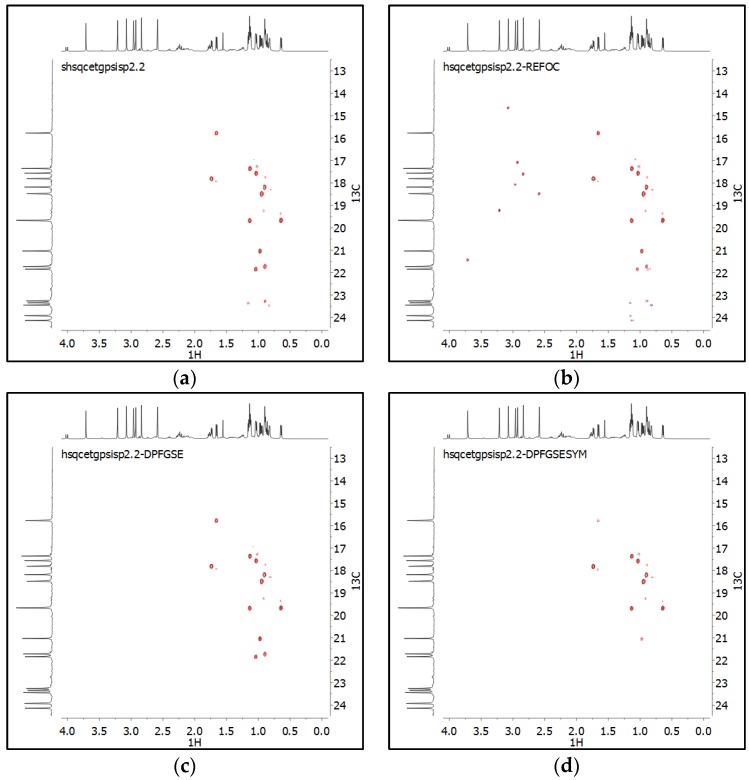
Bs-HSQC spectra recorded for CH_3_ γ Me-Val 11 signal of the cyclosporin sample, using the same ^13^C selective pulse (Q3.1000) in different sequences: shsqcetgpsisp2.2 (**a**); hsqcetgpsisp2.2-REFOC (**b**); hsqcetgpsisp2.2-DPFGSE (**c**); and hsqcetgpsisp2.2-DPFGSESYM (**d**).

Bs-HSQC spectra differed according to the sequence used (Figure 1): selectivity was much sharper for hsqcetgsisp2.2-DPFGSESYM, with only the signals resonating below 350 Hz from the ^13^C carrier frequency being visible. It can be noticed that, for the hsqcetgpsisp2.2-REFOC sequence, selectivity was also sharp, as the signals of carbons resonating more than 500 Hz away from the ^13^C carrier frequency are not pure phase, but unwanted signals (intense CH_3_N signals resonating from δ 2.5 to 3.8 ppm in ^1^H) were observed as aliased signals. These artifacts certainly are due to 180° pulse imperfection, since band-selective pulse powers were calculated and not manually calibrated. Miscalibration of the selective 180° pulse could be compensated by bracketing gradients in other pulse sequences, but not in this one.

It can be seen in Figure 1 that all HSQC experiments were sensitive and revealed minor compounds that were difficult to observe in ^1^H or ^13^C spectra (e.g., δ^13^C 19.2 ppm and δ^1^H 0.91 ppm).

For HMBC spectra, the two sequences (shmbcctetgpl2nd and hmbcctgpl2nd-SPFGSE) gave comparable spectra, except for a 20% sensitivity loss for the sequence including SPFGSE. The increased sequence duration due to SPFGSE insertion probably induces significant magnetization losses due to relaxation.

### 3.2. Selectivity According to Carbon Multiplicity

**Figure 2:**
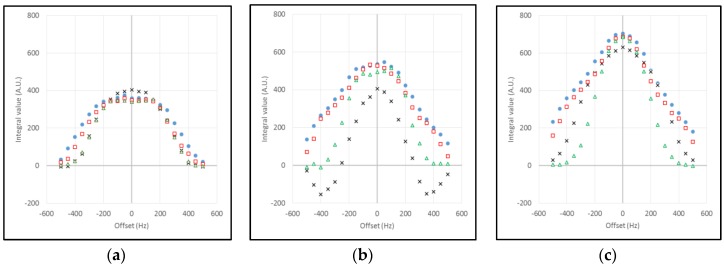
Influence of the ^13^C selective pulse offset on the bs-HSQC signal for three representative signals of cyclosporin sample: CH α Me-Leu 10 (**a**); CH_2_ β Me-Leu 10 (**b**); and CH_3_ γ Me-Val 11 (**c**). Four series are plotted for the following sequences: shsqcetgpsisp2.2 (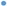); hsqcetgpsisp2.2-REFOC (×); hsqcetgpsisp2.2-DPFGSE (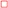); and hsqcetgpsisp2.2-DPFGSESYM (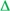). Selective pulse (Q3.1000) was calculated for a 600 Hz selectivity.

As formerly described [29], the selectivity profiles are modified by the action of one or more heteronuclear couplings: the more coupled ^1^H, the sharper the selectivity profile (Figure 2). Among the studied sequences, shsqcetgpsisp2.2 and hsqcetgisisp2.2-DGPGSE showed similar selectivity profiles. The symmetrical hsqcetgpsisp2.2-DPFGSESYM sequence, where a PFGSE is inserted before the t1 evolution period, is notably more selective than the non-symmetrical sequence (all theoretical selectivity profiles were calculated in the Nuzillard et al. paper [29]). An unexpected behavior of hsqcetgpsisp2.2-REFOC sequence can be seen in the case of CH_2_: outside from the sharp region where the observed peak is positive the integral value can become negative instead of just tending towards zero. This “oscillating” selectivity profile may explain the appearance of mix-phase or aliased signals for ^13^C resonating far from the carrier frequency.

For both bs-HMBC sequences, the integral value (measured on the Abu-CO peak at 173 ppm) showed a selectivity profile quite flat from −200 to +200 Hz (Supplementary Materials, Figure S3).

### 3.3. Influence of INEPT Interpulse Delay Setting

HSQC related sequences are constituted of two INEPT blocks: the first INEPT transfers magnetization from ^1^H to ^13^C, and after the t_1_ evolution period, magnetization is transferred back to ^1^H by a reverse INEPT. The interpulse delay of these INEPT blocks is usually set to 1/4*J* with *J* = 145 Hz, a commonly admitted mean value for ^1^*J*_CH_.

**Figure 3:**
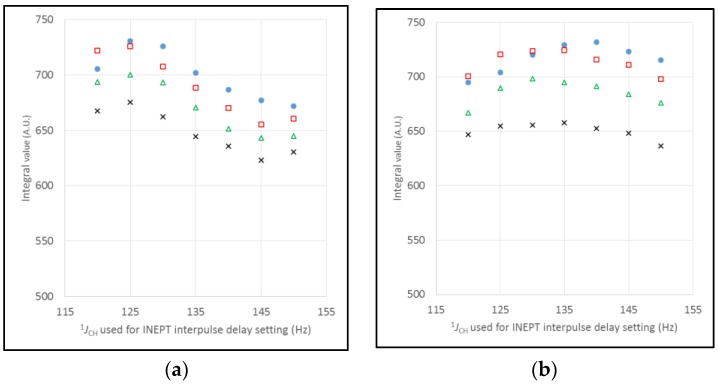
Influence of the chosen ^1^*J*_CH_ value for the INEPT interpulse delay setting on the bs-HSQC signal for two representative signals of cyclosporin sample: CH_3_ γ Me-Val 11 (^1^*J*_CH_ = 125Hz) (**a**); and CH_3_N Me-Leu 10 (^1^*J*_CH_ = 139 Hz) (**b**). Four series are plotted for the following sequences: shsqcetgpsisp2.2 (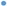); hsqcetgpsisp2.2-REFOC (×); hsqcetgpsisp2.2-DPFGSE (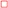); and hsqcetgpsisp2.2-DPFGSESYM (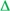).

It can be seen in Figure 3a that the integral value of CH_3_ γ Me-Val 11 signal of cyclosporin clearly was maximum when the interpulse delay was set with the accurate value ^1^*J*_CH_ = 125 Hz. All four bs-HSQC sequences showed the same dependence to ^1^*J*_CH_ value. For CH_3_N Me-Leu 10 signal, the optimum value should be ^1^*J*_CH_ = 139 Hz, but this was only visible for the shsqcetgpsisp2.2 sequence; for the three others, the optimum value seems to be ca. 130–135 Hz. For both studied signals, the most intense integral values were obtained for shsqcetgpsisp2.2 and hsqcetgpsisp2.2-DPFGSE sequences.

### 3.4. Relaxation Delay D1

Relaxation delay D1 is a long recycle delay (some seconds of duration, according to the existing T_1_^1^H relaxation times) to allow the recovery of the ^1^H magnetization to a pre-equilibrium state just before starting the sequence.

**Figure 4:**
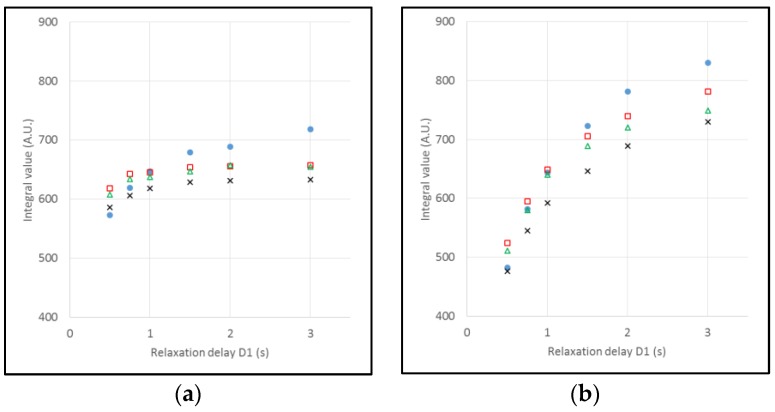
Influence of the relaxation delay D1 on the bs-HSQC signal for two representative signals of cyclosporin sample: CH_3_ γ Me-Val 11 (T_1_^1^H = 0.37 s) (**a**); and CH_3_N Me-Leu 10 (T_1_^1^H = 0.86 s) (**b**). Four series are plotted for the following sequences: shsqcetgpsisp2.2 (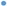); hsqcetgpsisp2.2-REFOC (×); hsqcetgpsisp2.2-DPFGSE (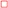); and hsqcetgpsisp2.2-DPFGSESYM (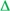).

As expected, integral values of bs-HSQC signals variations relative to the relaxation delay D1 used in the sequence are much more important for a slow relaxing ^1^H (CH_3_N Me-Leu 10, T_1_^1^H = 0.86 s, see Figure 4b) than for a rapid relaxing ^1^H (CH_3_ γ Me-Val 11, T_1_^1^H = 0.37 s, Figure 4a). It can be noticed that the sequence shsqcetgpsisp2.2 seems to be more sensitive in both cases to this relaxation delay effect than the other three.

For bs-HMBC sequences, relaxation delay effect is averaged because plotted signals are the sum of correlation peak’s integrals obtained for slow and quick relaxing ^1^H (Supplementary Materials, Figure S4).

### 3.5. Application to the Microstructural Determination of PHB^OAll^

As previously reported, the stereoselective ROP of *rac*-BPL^OAll^ by diamino-bis(phenolate) yttrium catalyst provides different microstructures of PHB^OAll^ depending on the *ortho*-substituent group on the ligand [21] (Scheme 1), as assessed by ^13^C{^1^H} analysis.

**Figure 5:**
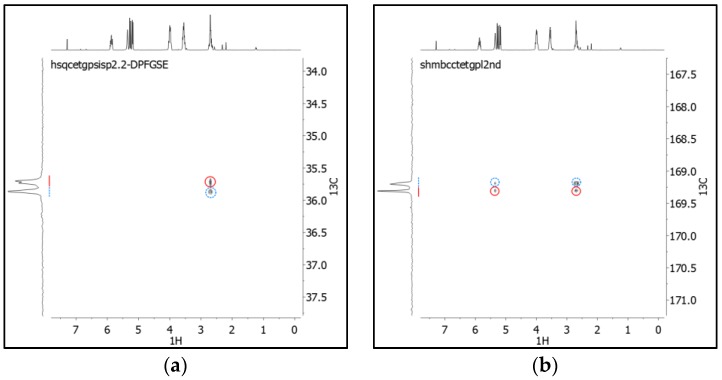
Integration regions for the *syndiotactic* (solid red line) and *isotactic* (dot blue line) signals of a PHB^OAll^ sample prepared with a non-stereoselective complex (**1a**, R = Me; *P*_s_ ≈ *P*_i_ ≈ 0.5). Side chain CH_2_ region observed by bs-HSQC (**a**) and carbonyl region observed by bs-HMBC (**b**).

The probability of *syndiotactic* enchainment between BPL^OR^ units, *P*_s_ (note: *P*_s_ = 1 − *P*_i_, *P*_i_ = probability of *isotactic* enchainment), is determined as follows:

*P*_s_ = *I*_s_/(*I*_s_ + *I*_i_), (1)

with *I*_s_ and *I*_i_ being the integral values of peaks assigned to *syndiotactic* or *isotactic* diads, respectively. Figure 5 shows the regions of the 1D ^13^C{^1^H}, bs-HSQC and bs-HMBC spectra and the integration regions of a PHB^OAll^ sample prepared with a non-stereoselective catalyst (**1a**, R = Me; *P*_s_ ≈ *P*_i_ ≈ 0.5).

For all spectra, precision of *P*_s_ determination is anticipated to decline when the tacticity tends to be highly *syndiotactic* or *isotactic*, because one of the two integrated signals will necessarily have a poor S/N ratio. As can be seen in Table 3, this is probably the case, but with only three replicates per measurement, standard deviation can sometimes be incidentally low.

It can be noticed that the *P*_s_ value obtained from 1D ^13^C{^1^H} can differ according to the observed region (carbonyl or side chain CH_2_). This seems to be the case when *P*_s_ is higher, maybe because additional signals which could be assigned to triads become visible when the “*iso*” peak decreases, and the integration limits are therefore no longer suitable in both regions.

For 1D ^13^C{^1^H} and HSQC, the comparison of integral values from *syndiotactic* and *isotactic* signals can be readily made, because they differ only in the relative stereochemistry of nearby nuclei. Carbon multiplicity being identical, relaxation rates and ^1^*J*_CH_ are expected to be very similar and off-resonance effect are negligible since the two peaks are resonating very close from each other, and the ^13^C carrier frequency is set in the middle. Indeed, as can be seen in Table 3, *P*_s_ values calculated from bs-HSQC sequences and 1D ^13^C{^1^H} for the 35 ppm region were similar. Standard deviations were satisfactory (<1.5%, <1% in most cases) for all sequences. The total acquisition time was 106 min for the 1D ^13^C{^1^H} and 37 min for the HSQC experiments. Note that we used the non-selective HSQC sequence in addition to the four bs-HSQC sequences, because in this compound no aliased signal can interfere with the integral value measurement of the studied signal, since δ^1^H are really different.

**Figure 6:**
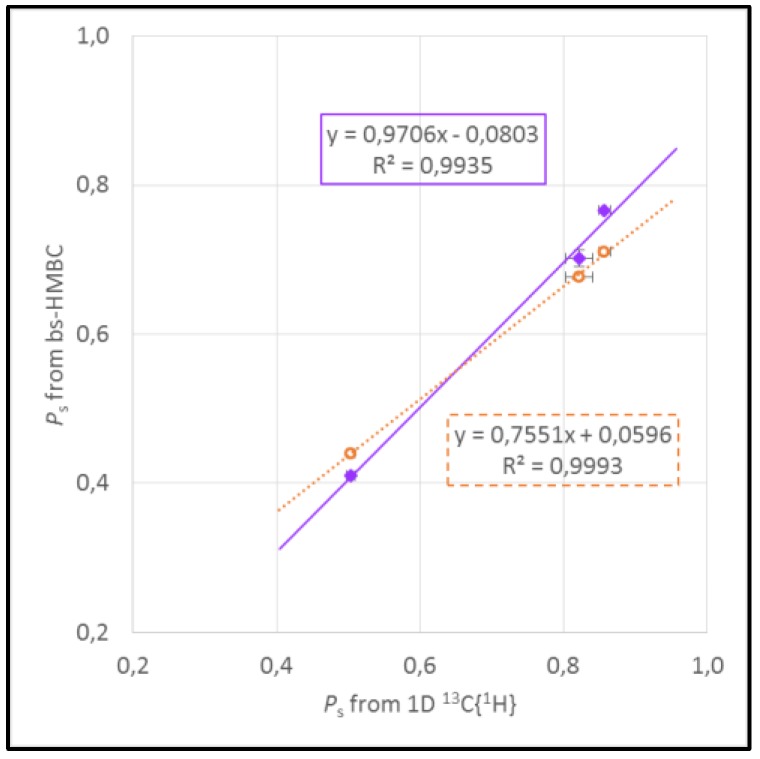
Plot of *P*_S_ values of PHB^OAll^ samples prepared with complexes **1a**–**c**, obtained from bs-HMBC with the shmbcctetgpl2nd sequence (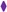) and with the hmbcctetgpl2nd-SPFGSE sequence (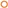) against *P*_S_ values obtained from 1D ^13^C{^1^H} (carbonyl region). Error bars represent standard deviation.

For bs-HMBC sequences, the *P*_s_ values obtained from bs-HMBC (40 min duration) really differed from those obtained from the carbonyl region of 1D ^13^C{^1^H}. A possible explanation could be small variations of ^n^*J*_CH_ involved in HMBC correlations between the various stereo-configurations (whereas ^1^*J*_CH_ values involved in HSQC are quite stable), inducing differences in the relative integral values measured for *syndiotactic* or *isotactic* signals. However, we noticed that, for the three studied PHB^OAll^ samples (prepared with complexes **1a**–**c** bearing R = Me, *t*Bu and CMe_2_Ph substituents, respectively; Scheme 1), a linear relation between the two sets of *P*_s_ values could be considered (Figure 6). Standard deviations were satisfactory, maybe even better for bs-HMBC sequences than for 1D ^13^C{^1^H} (slow relaxing carbonyl signals have a lower S/N ratio than CH_2_, and this sensitivity effect could be compensated by the ^1^H detection of multiple signals in bs-HMBC).

## 4. Discussion

As expected, methodological study on the cyclosporin sample showed that bs-HSQC signal intensity is really dependent of carbon multiplicity, relaxation delay and (to a lower extent) INEPT interpulse delay setting: this will prevent in most cases the use of such sequences for quantitative purposes. When similar signals are to be compared, however, bs-HSQC can provide a shorter duration alternative to 1D ^13^C{^1^H}, even in cases with small δ^13^C dispersion.

For bs-HSQC sequences, all methods used for introducing selectivity are not technically quite the same: selectivity is sharper for the sequence using symmetric DPGFSE (hsqcetgpsisp2.2-DPFGSESYM), and for the observation of CH_3_ signals, off-resonance effects must be taken in account, which can induce a 50% signal loss for only a 200 Hz offset when the selective pulse had been designed for a 600 Hz bandwidth. Selectivity observed for standard Bruker shsqcetgpsisp2.2 and for the sequence including a DPFGSE (hsqcetgpsisp2.2-DPFGSE) after t_1_ evolution period, even if dependent on carbon multiplicity, is consistent with the bandwidth value chosen for selective pulse design. In contrast, sequence using a selective refocalization pulse in the reverse INEPT step (hsqcetgpsisp2.2-REFOC) showed a peculiar selectivity profile, with a sharp region of pure phase signals, mixed phase signals for peaks resonating on the edges of the selective pulse bandwidth and even unwanted aliased signals for intense peaks resonating far away. This could be due to 180° selective pulse miscalibration, not compensated in this pulse sequence because of the absence of bracketing gradients. Aliasing of signals resonating outside of the chosen selectivity zone is obviously a problem for both quantification applications (in case an aliased signal would be superimposed with a studied signal) and structural studies where bs-HSQC is used for improving ^13^C resolution. This problem does not seem to have been reported by previous authors using this selectivity method [31,32,33,34].

All four bs-HSQC sequences showed similar dependence to the ^1^*J*_CH_ value chosen for INEPT interpulse delay setting. Variations of signal integral value observed when varying relaxation delay D1 were also equivalent for three of them, with a loss of sensitivity induced using a too short delay. This, as expected, is more important for slow relaxing hydrogens. We can note that the standard Bruker sequence shsqcetgpsisp2.2 is more sensitive to this relaxation delay effect. Consequently, this sequence should be avoided for a quantitative purpose when large variations of relaxation times are observed in the compound.

For bs-HMBC, the only difference observed between the two sequences (selectivity obtained by a selective refocalization pulse or by a SPFGSE included after the t_1_ evolution period) is a decrease of 20% in sensitivity for the longer sequence (hmbcctetgpl2nd-SPFGSE), probably due to magnetization losses owing to relaxation.

Concerning the application of bs-HSQC sequences for determining tacticity of alkoxy-functionalized poly(3-hydroxyalkanoate)s (PHB^OR^s), the method’s accuracy seems satisfactory since the *P*_s_ values obtained from bs-HSQC acquisitions were very similar to those obtained from 1D ^13^C{^1^H} on the same region (side-chain CH_2_). We ensured that a faster acquisition (37 or 40 min vs. 106 min for 1D ^13^C{^1^H} spectrum, respectively) did not lead to a loss of measurements precision. The reduction in time to accurately determine the tacticity of the PHB^OR^s using this bs-HSQC experiment is of the same extent as that reported by Coates and co-workers. [22] Indeed, the pentad stereosequences of atactic polypropylene and the triad stereosequences of atactic polyacrylonitrile were determined in 20 and 9 min from the 2D bs-HSQC, as compared to the 270 and 56 min total experiment time using the quantitative direct-observed ^13^C NMR spectrum, respectively, on a similar narrow chemical shift window [22]. The observed standard deviations were very satisfactory, with a precision of about 0.1% on *P*_s_ determination. This result was obtained on challenging compounds, since the *syndiotactic* and *isotactic* signals are only separated by about 0.2 ppm. No significant difference could be made between the four bs-HSQC sequences for this study of PHB^OR^s tacticity.

Bs-HMBC could be useful for tacticity studies when only the carbonyl ^13^C signals are resolved enough for discriminating between *syndiotactic* and *isotactic* configurations. However, this experiment cannot be used straightforwardly for *P*_s_ determination: we believe that changes in bond angles, induced by stereochemistry differences, give rise to variations of ^n^*J*_CH_ values involved in HMBC, and that such variations affect signal intensities between *syndiotactic* and *isotactic* configurations. A linear relation between *P*_s_ obtained from bs-HMBC and *P*_s_ obtained from 1D ^13^C{^1^H} of the same region (carbonyl) was observed for polymer samples prepared from complexes **1a**–**c**. This linear relation would need to be confirmed in a future work. If so, for a given polymer, a calibration could be performed with samples of varying tacticity, allowing the use of bs-HMBC sequence for further polymer tacticity screening (e.g., when testing catalysts for stereoregular polymerization). It can be pointed out that standard deviations of *P*_s_ values obtained from bs-HMBC sequences were smaller than those of *P*_s_ values obtained from 1D ^13^C{^1^H} spectra; in this case, the fastest acquisition seems to be also the most precise.

## 5. Conclusions

In conclusion, we have described the use of 2D bs-HSQC and bs-HMBC NMR experiments as a technique for rapidly evaluating the tacticity of some PHBs. The methodological study on cyclosporin showed a better selectivity profile and a lower sensitivity to relaxation delay shortening for bs-HSQC sequence including a DPFGSE as ^13^C selectivity scheme and we are thus confident this is the suitable sequence for quantitative applications. These methods can be applied with a good accuracy to resolve high-order stereosequences as exemplified with the triads of some poly(3-hydroxyalkanoate)s. A significant acquisition time benefit can be gained while maintaining a high resolution, ultimately enabling *P*_s_ determination with a precision of about 0.1%. We believe the bs-HSQC NMR tool may be valuably developed for the routine and rapid recording of well-resolved narrow-spectral window ^13^C NMR spectra, as well as for the general assessment of the tacticity of polymers. Future work will address the larger exploration of the bs-HMBC NMR tool on related polymers.

## Figures and Tables

**Table 1:** Repeatability of integral value measurement for the CH_3_ γ Me-Val 11 peak of cyclosporin (with selective pulse offset = 0 Hz, relaxation delay D1 = 1.5 s, INEPT interpulse delay set for *J* = 145 Hz).

NMR Sequence	Number of Measurements	Mean Integral Value (A.U.)	Standard Deviation of Integral Value (A.U.)	Relative Standard Deviation (%)
shsqcetgpspsp2.2	7	688.2	15.0	2.2%
hsqcetgpsisp2.2-REFOC	6	622.5	10.4	1.7%
hsqcetgpsisp2.2-DPFGSE	7	666.1	15.6	2.3%
hsqcetgpsisp2.2-DPFGSESYM	6	654.7	15.7	2.4%

**Table 2:** Selected peaks of cyclosporin.

Attribution	δ^13^C	δ^1^H	T_1_^13^C	T_1_^1^H	^1^ *J*_CH_
Abu-CO	173.71	-	1.44	-	-
CH α Me-Leu 10	57.84	5.34	0.29	0.38	134
CH_2_ β Me-Leu 10	41.55	2.41/1.29	0.17	0.40	126/132
CH_3_N Me-Leu 10	29.98	2.84	1.13	0.86	139
CH_3_ γ Me-Val 11	18.87	0.96	0.28	0.37	125

**Table 3:** *P*_s_ (probability of *syndiotactic* enchainment between BPL^OAll^ units) determined by different NMR sequences on PHB^OAll^ samples prepared with complexes **1a**–**c** (bearing R = Me, *t*Bu and CMe_2_Ph substituents, respectively; Scheme 1). Each measurement was replicated three times for mean and standard deviation calculation.

Complex Used to Prepare PHB^OAll^	Sequence	Observation of Carbonyl Signal (δ 169 ppm)		Observation of Side Chain CH_2_ (δ 35 ppm)	
		*P*_s_ (Mean)	S.D.	*P*_s_ (Mean)	S.D.
**1a**	zgpg	0.504	0.006	0.504	0.004
	hsqcetgpsisp2.2			0.499	0.001
	shsqcetgpsisp2.2			0.503	0.003
	hsqcetgpsisp2.2-REFOC			0.507	0.005
	hsqcetgpsisp2.2-DPFGSE			0.501	0.007
	hsqcetgpsisp2.2-DPFGSESYM			0.503	0.003
	shmbcctetgpl2nd	0.410	0.002		
	hmbcctetgpl2nd-SPFGSE	0.440	0.004		
**1b**	zgpg	0.822	0.019	0.784	0.001
	hsqcetgpsisp2.2			0.795	0.007
	shsqcetgpsisp2.2			0.797	0.014
	hsqcetgpsisp2.2-REFOC			0.800	0.011
	hsqcetgpsisp2.2-DPFGSE			0.791	0.004
	hsqcetgpsisp2.2-DPFGSESYM			0.827	0.011
	shmbcctetgpl2nd	0.702	0.012		
	hmbcctetgpl2nd-SPFGSE	0.676	0.002		
**1c**	zgpg	0.857	0.008	0.812	0.004
	hsqcetgpsisp2.2			0.837	0.005
	shsqcetgpsisp2.2			0.834	0.008
	hsqcetgpsisp2.2-REFOC			0.839	0.005
	hsqcetgpsisp2.2-DPFGSE			0.828	0.001
	hsqcetgpsisp2.2-DPFGSESYM			0.863	0.009
	shmbcctetgpl2nd	0.766	0.001		
	hmbcctetgpl2nd-SPFGSE	0.711	0.003		

## Supplementary Materials

The following are available online at http://www.mdpi.com/2073-4360/10/5/533/s1, Figure S1: HSQC sequences: Standard Bruker hsqcetgpsisp2.2, Figure S2: HMBC sequences: Standard Bruker hmbcctetgpl2nd, Figure S3: Influence of the ^13^C selective pulse offset on the bs-HMBC signal of Abu-CO in cyclosporin sample, Figure S4: Influence of the relaxation delay D1 on the bs-HMBC signal of Abu-CO in cyclosporin sample, Figure S5: Full ^13^C{^1^H} spectrum of a PHB^OAll^ sample, Figures S6–S8: ^1^H NMR spectra of PHB^OAll^ samples prepared from the ROP of *rac*-BPL^OAll^ with the **1a**–**c**/*i*PrOH (1:1) systems.

## Abbreviations

BPL^OR^	alkoxymethylene-β-propiolactones
bs	band-selective
DANTE	delays alternating with nutation for tailored excitation
DPFGSE	double pulsed field gradients spin-echo
GARP	globally-optimised, alternating phase rectangular pulses
HMBC	heteronuclear multiple bond correlation
HSQC	heteronuclear single quantum correlation
INEPT	insensitive nuclei enhanced by polarization transfer
MLA^R^	alkyl β-malolactonate
PHB^OR^	poly(3-hydroxyalkanoate)
PLA	polylactide
ROP	ring-opening polymerization
