# Supplementary material for: Evaluation of Band-Selective HSQC and HMBC: Methodological Validation on the Cyclosporin Cyclic Peptide and Application for Poly(3-hydroxyalkanoate)s Stereoregularity Determination
Source: Polymers (Basel). 2018 May 16;10(5):533. doi: 10.3390/polym10050533 (PMC6415406; doi:10.3390/polym10050533)
Supplement: Supplementary file 1 [file polymers-10-00533-s001.docx]

Supplementary Materials: Evaluation of Band-Selective HSQC and HMBC: Methodological Validation on the Cyclosporin Cyclic Peptide and Application for Poly(3-hydroxyalkanoate)s Stereoregularity Determination

Elsa Caytan ^1,^*, Romain Ligny ^1^, Jean-François Carpentier ^1^, and Sophie M. Guillaume ^1^

^1^ Univ Rennes, CNRS, Institut des Sciences Chimiques de Rennes – UMR6226, F-35000 Rennes, France

***** Correspondence: elsa.caytan@univ-rennes1.fr, Tel.: +33 2 23 23 73 93

| (**a**) | 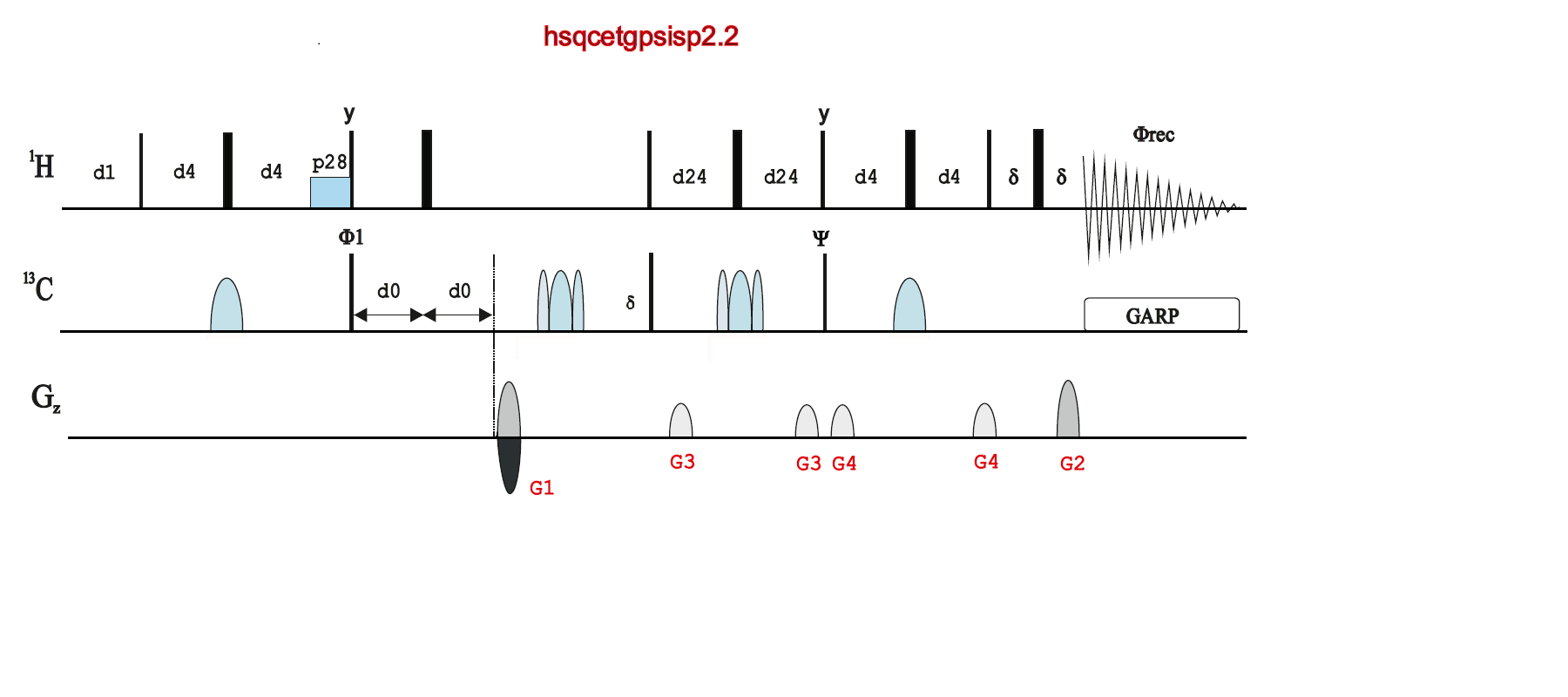 |
| --- | --- |
| (**b**) | 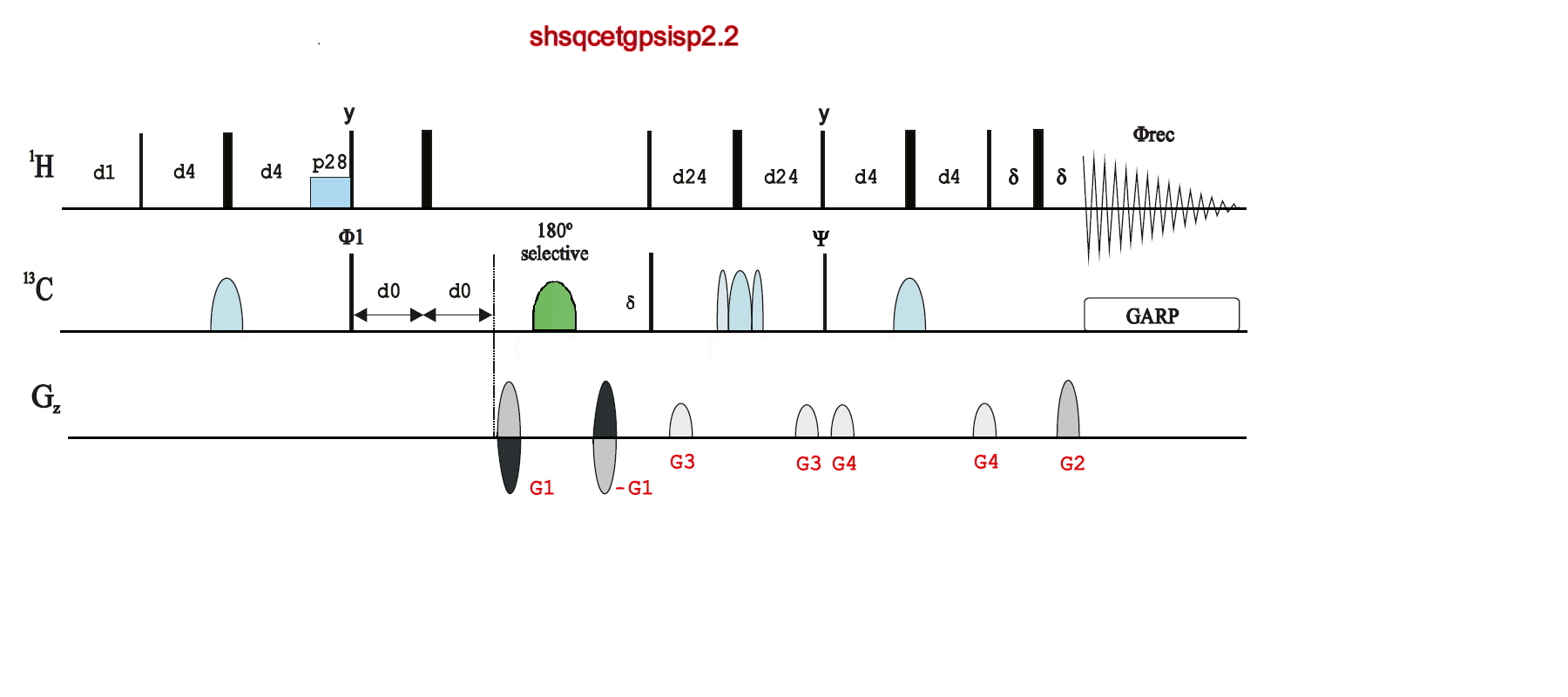 |
| (**c**) | 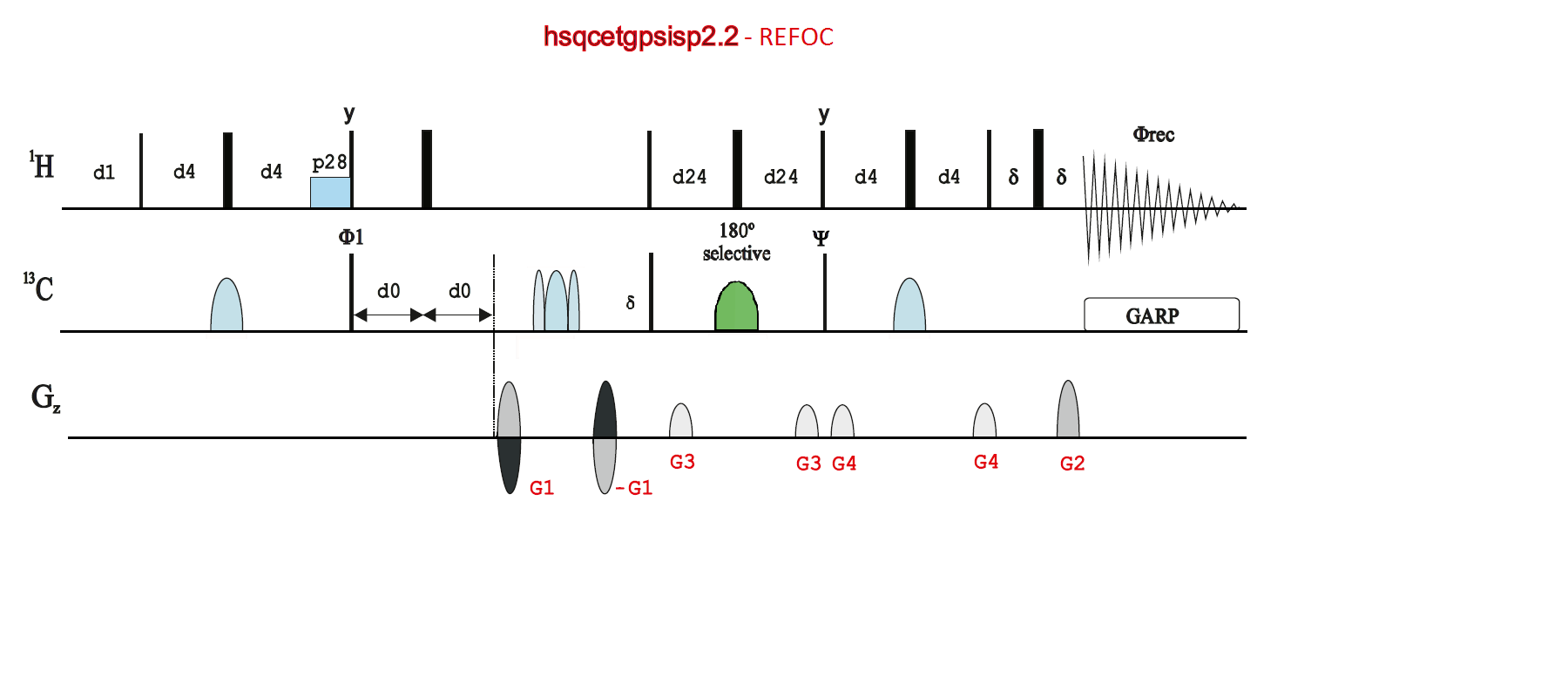 |
| (**d**) | 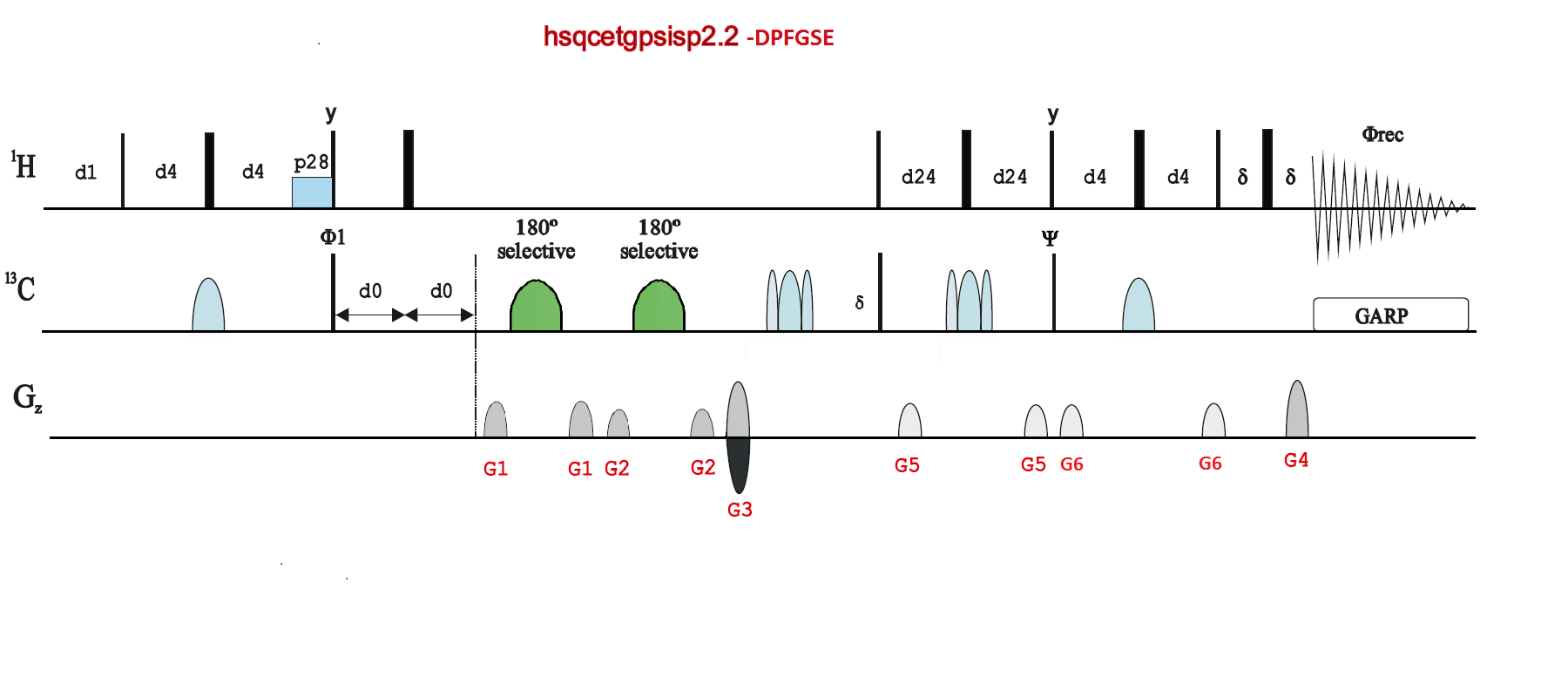 |
| (**e**) | 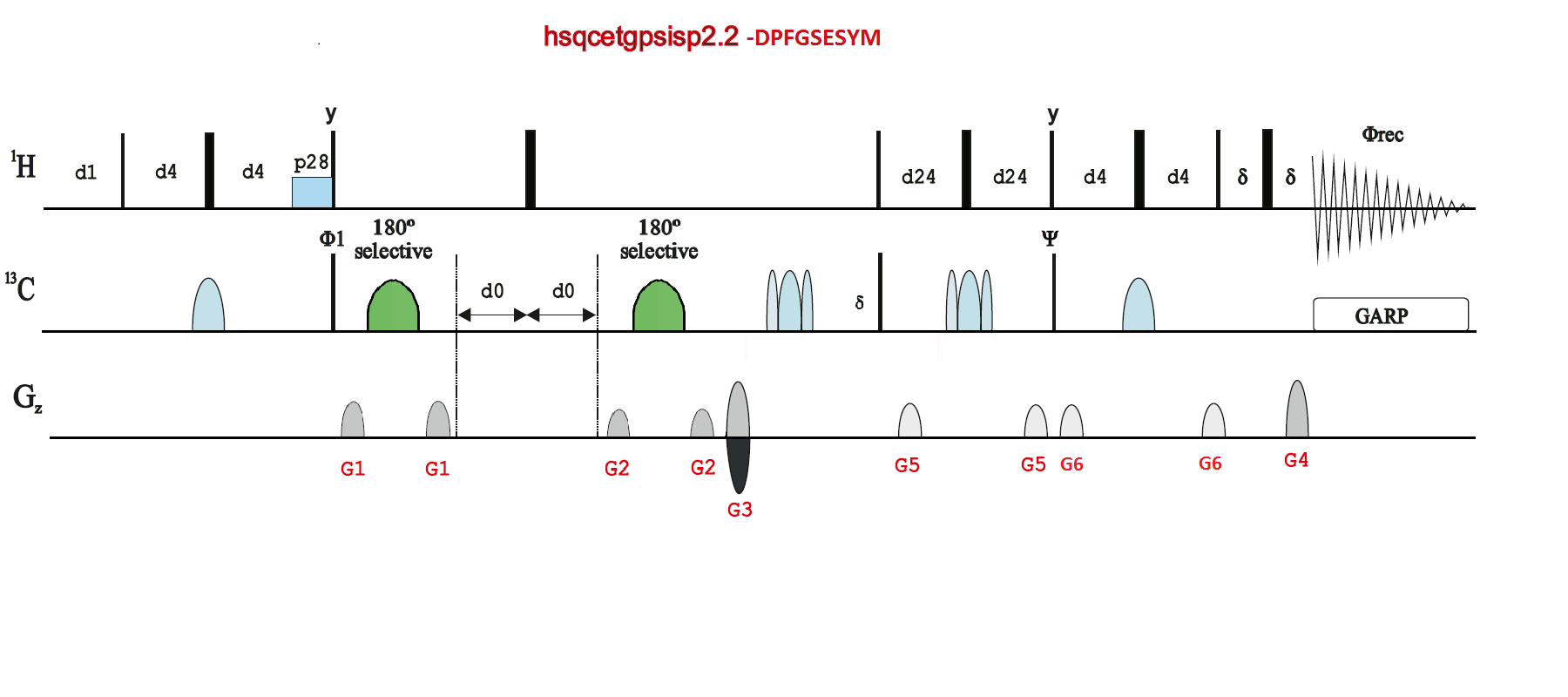 |

**Figure S1.** HSQC sequences: Standard Bruker hsqcetgpsisp2.2 (a); Standard Bruker shsqcetgpsisp2.2 with first ^13^C adiabatic refocalisation pulse replaced by a selective 180° refocalisation pulse bracketed by gradients of opposite sign (b); hsqcetgpsisp2.2-REFOC with ^13^C adiabatic refocalisation of the reverse INEPT replaced by a selective 180° refocalisation pulse (c); hsqcetgpsisp2.2-DPFGSE with a double pulsed field gradients spin-echo (DPFGSE) using selective 180° inversion pulse added after the t1 evolution period (d); hsqcetgpsisp2.2-DPFGSESYM with one pulsed field gradients spin-echo (PFGSE) using selective 180° inversion pulse inserted before the t1 evolution period and another one after t1 evolution period. In all pulse sequences thin and thick vertical black bars indicate 90° and 180° pulses, respectively, blue shaped pulses are 180° adiabatic pulses (smooth Chirp or composite smoothed Chirp) and green shaped pulses are 180° selective pulse (Q3.1000 shape here).

| (**a**) | 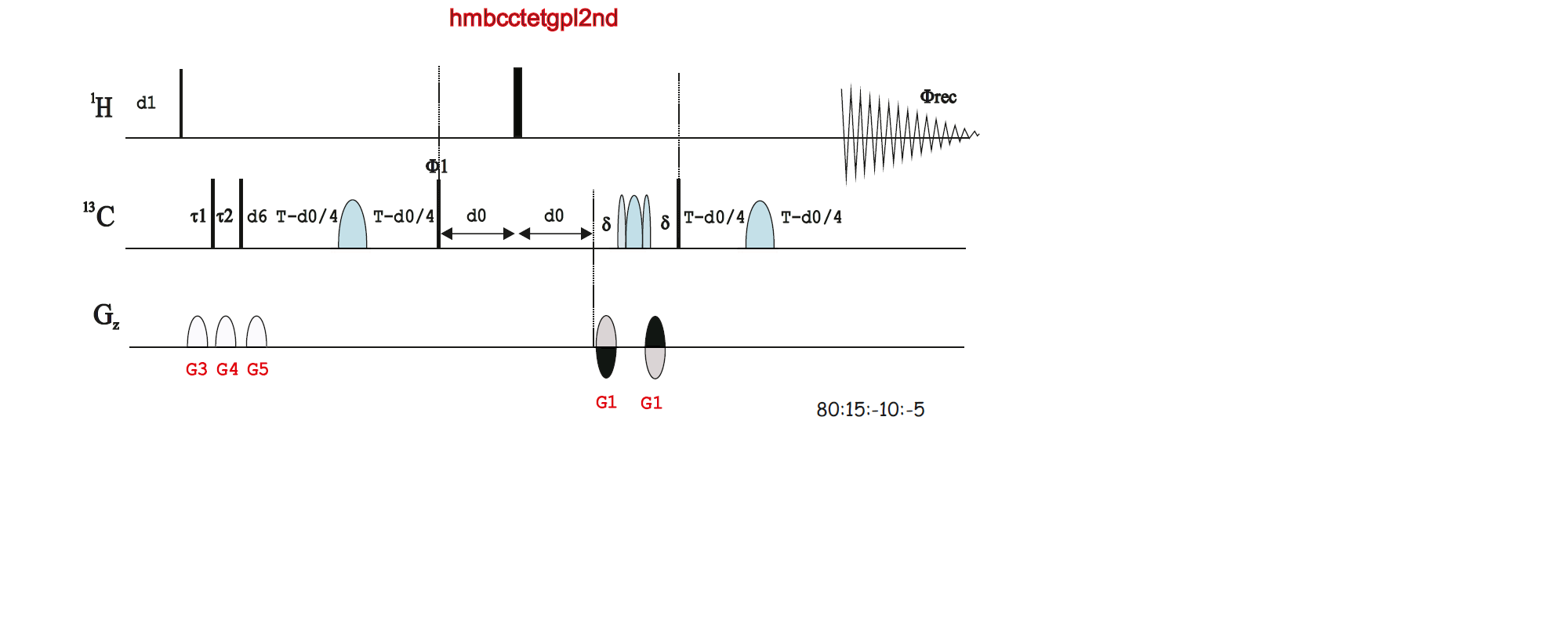 |
| --- | --- |
| (**b**) | 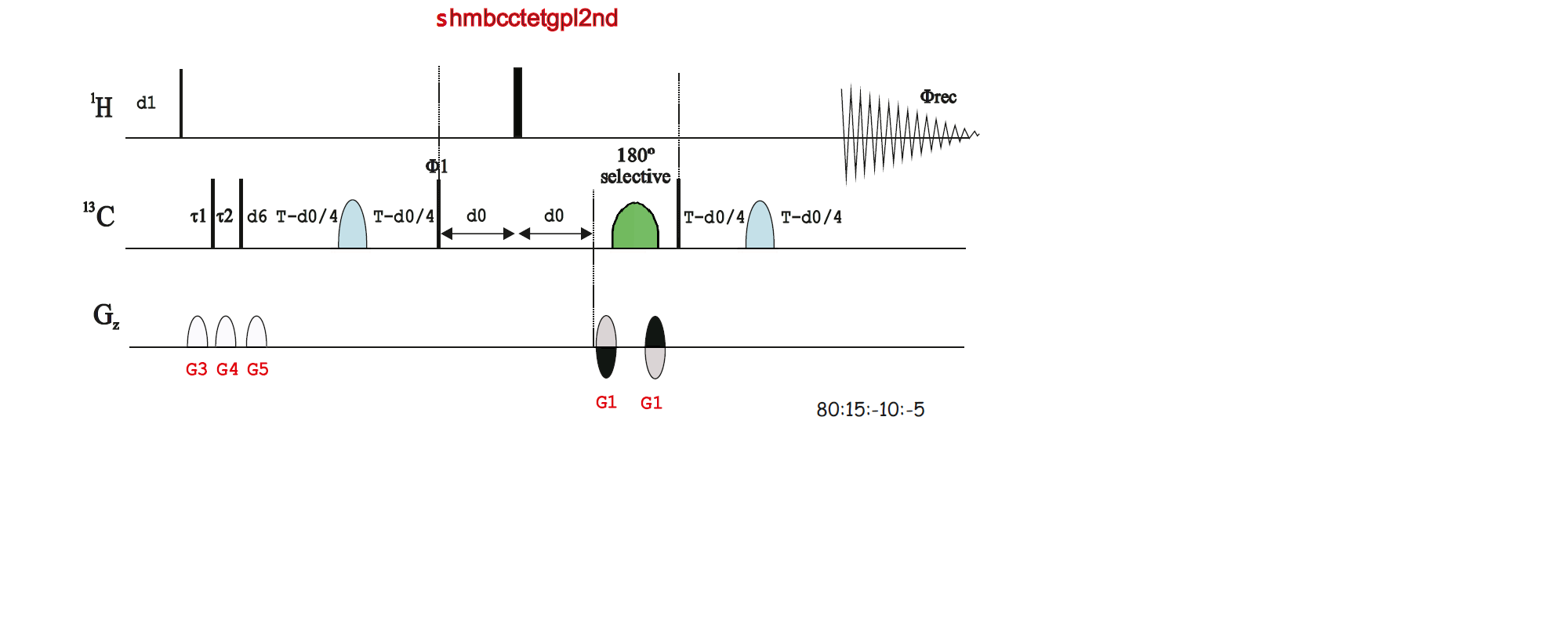 |
| (**c**) | 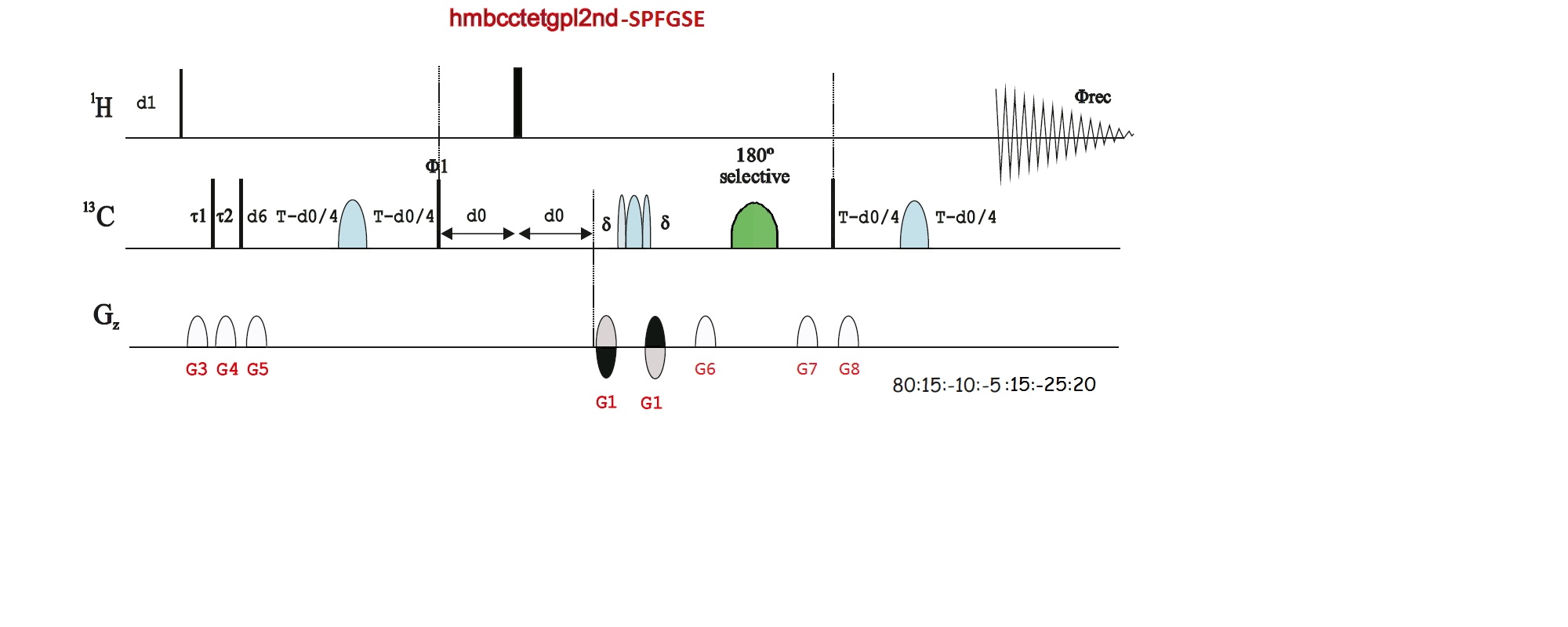 |

**Figure S2.** HMBC sequences: Standard Bruker hmbcctetgpl2nd (a); standard Bruker shmbcctetgpl2nd with ^13^C adiabatic refocalisation replaced by a selective 180° refocalisation pulse (b); hmbcctetgpl2nd-SPFGSE with a single pulsed field gradient spin-echo (SPFGSE) using selective 180° inversion pulse inserted after the t1 evolution period. In all pulse sequences thin and thick vertical black bars indicate 90° and 180° pulses, respectively, blue shaped pulses are 180° adiabatic pulses (smooth Chirp or composite smoothed Chirp) and green shaped pulses are 180° selective pulse (Q3.1000 shape here).

| 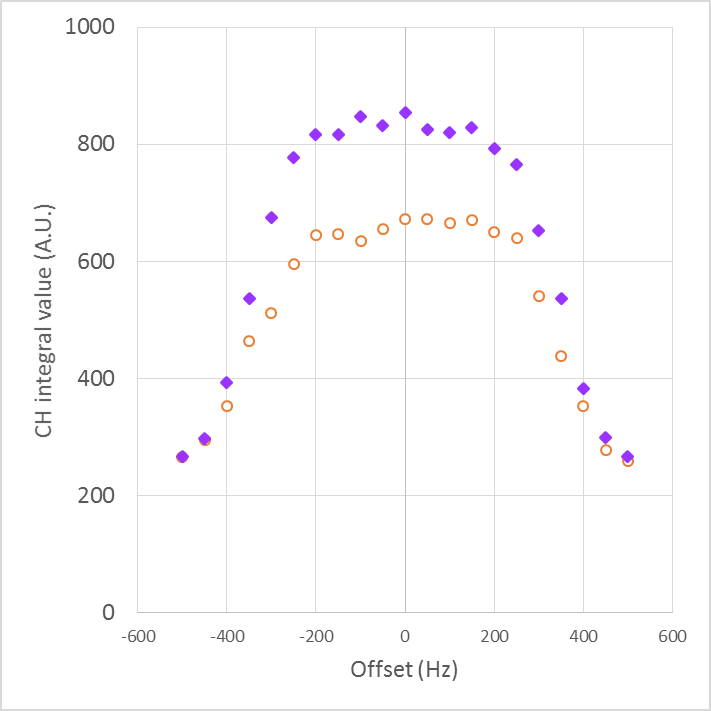 |
| --- |

**Figure S3.** Influence of the ^13^C selective pulse offset on the bs-HMBC signal of Abu-CO in cyclosporin sample. Two series are plotted for the following sequences: shmbcctetgpl2nd (⯁); hmbcctetgpl2nd-SPFGSE (**🞅**). Selective pulse (Q3.1000) was calculated for a 600 Hz selectivity.

| 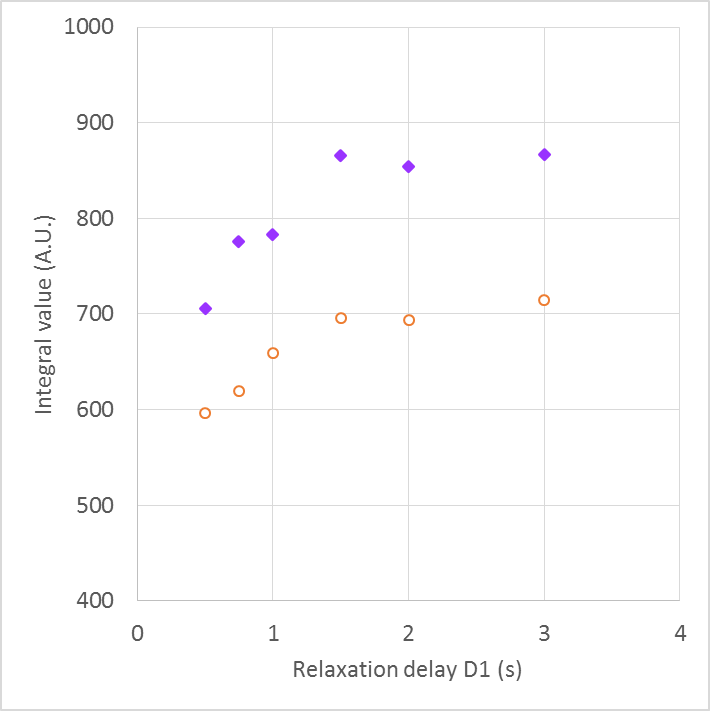 |
| --- |

**Figure S4.** Influence of the relaxation delay D1 on the bs-HMBC signal of Abu-CO in cyclosporin sample. Two series are plotted for the following sequences: shmbcctetgpl2nd (⯁); hmbcctetgpl2nd-SPFGSE (**🞅**).

| 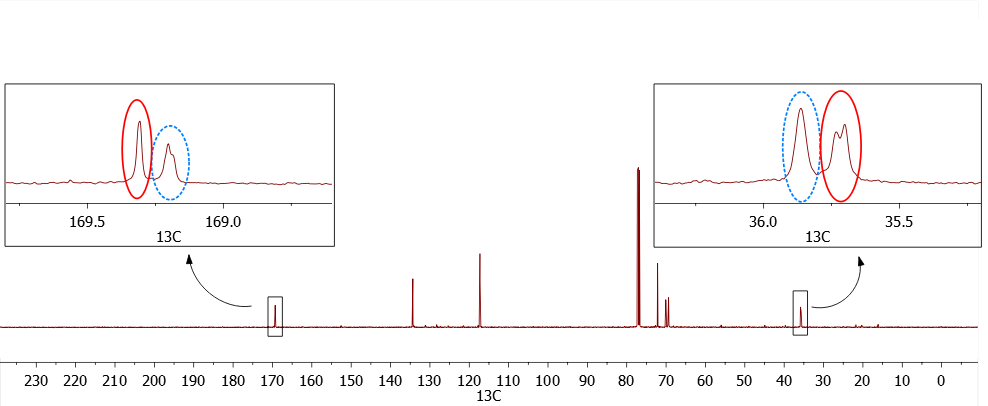 |
| --- |

**Figure S5.** Full ^13^C{^1^H} spectrum of a PHB^OAll^ sample prepared with a non-stereoselective catalyst (**1a**, R = Me; *P*_s_ ≈ *P*_i_ ≈ 0.5). *Syndiotactic* (solid red line) and *isotactic* (dot blue line) signals are highlighted.

|  |
| --- |

**
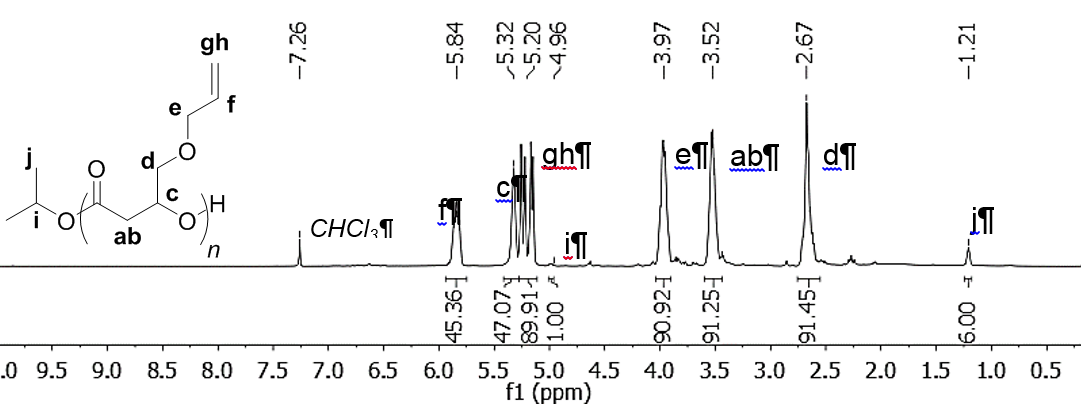
**

**Figure S6.** ^1^H NMR spectrum (500 MHz, CDCl_3_, 25 °C) of an *atactic* PHB^OAll^ prepared from the ROP of
*rac*-BPL^OAll^ with the **1a**/*i*PrOH (1:1) system (*P*_s_ = 0.50; Table 3).

|  |
| --- |

**
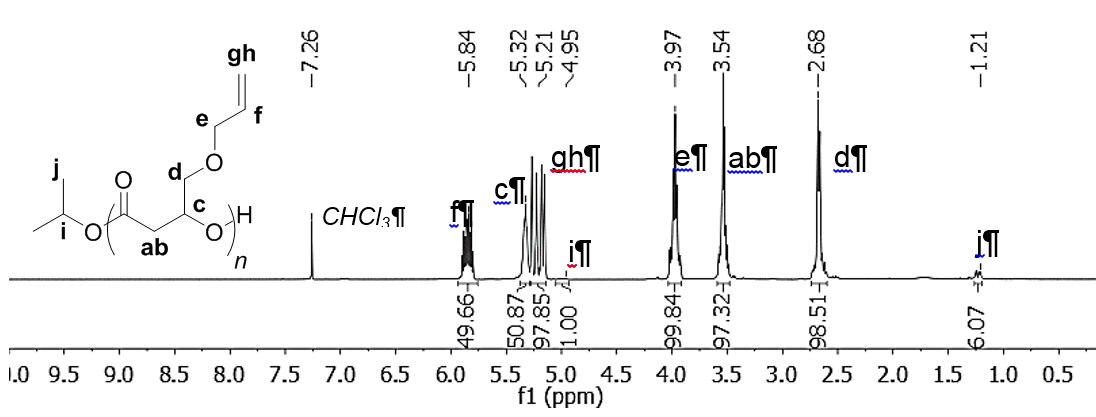
**

**Figure S7.** ^1^H NMR spectrum (400 MHz, CDCl_3_, 25 °C) of a *syndiotactic* PHB^OAll^ prepared from the ROP of *rac*-BPL^OAll^ with the **1b**/*i*PrOH (1:1) system (*P*_s_ = 0.82; Table 3).

|  |
| --- |

**
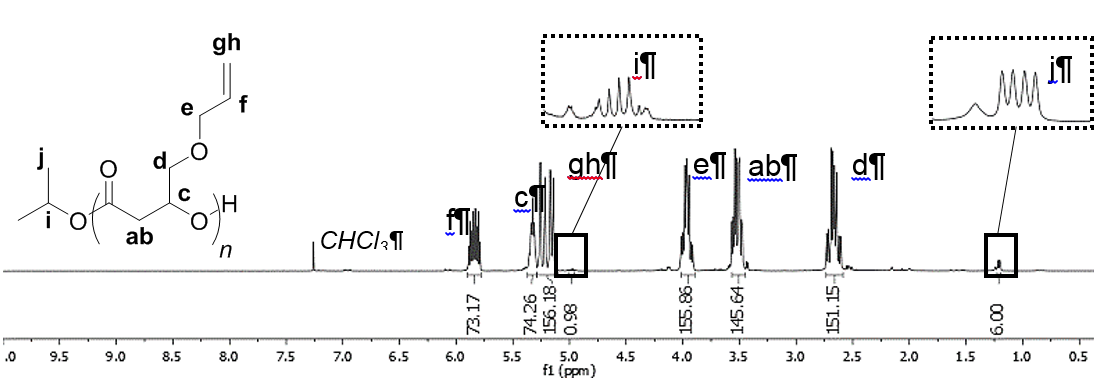
**

**Figure S8.** ^1^H NMR spectrum (500 MHz, CDCl_3_, 25 °C) of a *syndiotactic* PHB^OAll^ prepared from the ROP of *rac*-BPL^OAll^ with the **1c**/*i*PrOH (1:1) system (*P*_s_ = 0.86; Table 3).
